# The Hidden Influences: Psychological Drivers of Medical Practice Variation

**DOI:** 10.3390/jcm14207396

**Published:** 2025-10-20

**Authors:** Sagi Shashar, Moriah E. Ellen, Ehud Davidson, Shlomi Codish, Victor Novack

**Affiliations:** 1 Clinical Research Center, Soroka University Medical Center, Faculty of Health Sciences, Ben-Gurion University of the Negev, Beer-Sheva 84101, Israel; victorno@clalit.org.il; 2 Department of Health Policy and Management, Guilford Glazer Faculty of Business and Management, Faculty of Health Sciences, Ben Gurion University, Beer-Sheva 84105, Israel; ellenmo@bgu.ac.il; 3 Institute of Health Policy Management and Evaluation, Dalla Lana School of Public Health, University of Toronto, Toronto, ON M5T 3M6, Canada; 4 United Hatzalah, Jerusalem 36233, Israel; ehud.davidson@gmail.com; 5 Soroka University Medical Center, Faculty of Health Sciences, Ben-Gurion University of the Negev, Beer-Sheva 84101, Israel; shlomico@clalit.org.il

**Keywords:** medical practice variation, primary care, health services, MPV determinants, MPV explanation

## Abstract

**Background:** Previous research showed that the majority of the variation in providers’ practice patterns is unexplained by patient, physician, and primary care practice characteristics. This study assessed physicians’ personal behavioral characteristics as explanatory components of medical practice variation (MPV). **Methods:** In this cross-sectional study, primary care physicians from Clalit Health Services in southern Israel were interviewed using validated surveys assessing risk-taking, tolerance for ambiguity, stress due to uncertainty, fear of malpractice, and empathy. We analyzed how much these traits explained MPV compared to patient, physician demographic, occupational, and practice characteristics using generalized linear mixed models and Nakagawa’s R^2^. **Results:** Of the 160 physicians approached, 146 (91.3%) participated. The median practicing time was 22 years; 48% were male, with a median age of 49. The median number of patients per practice was 1135. Overall, 40.4% of MPV was explained, mostly by patient characteristics (18.9%), practice characteristics (10.2%), and physician demographics (8.3%). Physician behavioral traits explained only 2.3%. **Conclusions:** Personal behavior characteristics explain a minority of MPV, leaving 60% of the MPV unexplained. This suggests either limitations in survey assessments or that these traits are not key drivers of MPV.

## 1. Introduction

Medical practice variation (MPV)—the systematic differences in the use of tests, referrals, treatments, or procedures across clinicians or geographic areas that cannot be explained by patient need or preferences—has been documented for decades and is associated with potential overuse, underuse, and unwarranted inefficiencies in care [1,2]. Reducing unwarranted MPV is a health system priority because it reflects inconsistent quality and inefficient resource use; settings with greater unexplained variation often incur higher costs without better outcomes and can exacerbate inequities in access and care [3,4,5].

Framing MPV explicitly as a target for improvement clarifies why estimating its drivers is essential for policy and practice. The determinants of MPV are typically grouped into the following three categories: (a) patient characteristics (case mix, morbidity burden, preferences); (b) physician/provider characteristics (demographic, training/occupational factors, and—potentially—psychological/behavioral traits); and (c) practice context at the primary care practice level (e.g., practice type, panel size, patient-to-physician workload, and organizational features).

Our group has shown that MPV among primary care physicians is substantial and remarkably stable across years and services [6,7], and that patient- and practice-level factors explain a meaningful but incomplete portion of the variance in utilizations [7]. The present study constitutes the behavioral traits phase and is designed to complement our prior work [8]; while the earlier studies quantified the magnitude of MPV and apportioned variance to patient-, physician- (demographic/occupational), and practice-level domains [6,7], here we focus on whether the validated behavioral characteristics of physicians add explanatory power beyond those domains [9].

Previous research on psychological characteristics affecting physicians’ medical practice have included studies on personality types [10], risk attitudes [11], beliefs [12], adherence to treatment guidelines [13,14], empathy [15], and fear of malpractice [16]. These studies found associations between psychological characteristics and physicians’ utilization rates of different health services. Yet, most of these studies have not assessed the extent of the variation explained by these characteristics, or have not assessed them across diverse health services or throughout an extended period, and most have focused on a single characteristic.

The present study aimed to quantify the extent to which physicians’ validated behavioral and psychological characteristics—risk-taking, tolerance for ambiguity, stress due to uncertainty, fear of malpractice, and empathy—explain MPV in primary care. We focused on these five behavioral constructs because theory and prior empirical work link them directly to clinical decision-making under uncertainty and resource stewardship [15,17,18]. Each construct was measured with validated instruments developed for physicians, or widely used in physician populations, making them both behaviorally meaningful and methodologically appropriate [19]. We sought to determine the proportion of variance in health service utilization rates which was attributable to these behavioral traits, beyond what is explained by patient case mix, physician demographic and occupational characteristics, and practice-level context.

## 2. Methods

### 2.1. Study Design and Setting

In this cross-sectional study, we included primary care physicians who had been practicing in the non-private practices of Clalit Health Services in the southern region of Israel for longer than a year, during 2011–2017, and who had more than 100 adult patients per practice. Israel has universal coverage under the 1995 National Health Insurance Law. All residents enroll in one of four non-profit health plans (Kupot Holim) that finance and provide care in the community; plans operate under government regulation and a uniform statutory benefits basket. Clalit Health Services is an integrated payer–provider that owns/operates hospitals and a nationwide network of community primary care practices, and which is the largest (4.7 million insurers) of these non-profit plans and the largest healthcare provider in southern Israel, covering approximately 67% of its population of 800,000 residents. Primary care is predominantly delivered in multi-physician practices; physicians may practice in more than one practice within the health plan. Our sampling frame therefore included Clalit primary care physicians working in non-private (plan-owned) practices in southern Israel [20,21,22].

Israel is a high immigration country; consequently, a substantial share of physicians are foreign-born. Regardless of birthplace or training venue, all physicians practice under national licensing and accreditation standards. Foreign-trained physicians must meet the Ministry of Health requirements (including licensing examinations and internship/board equivalency) before independent practice. Residency curricula and board examinations are nationally standardized. Within Clalit, clinical protocols, professional development, and audit-and-feedback programs are applied uniformly across practices.

### 2.2. Data Collection

Data were obtained from the following two sources: (a) the computerized medical records of Clalit Health Services (patient and physician demographic, and occupational and practice data), and (b) supervised surveys. Computerized health data obtained for the analysis were described elsewhere [8]. In brief, the annual adult patient data for each physician per practice included demographic and health data (median age, percent male, marital status, practice area socioeconomic status, and percent with diabetes, hypertension, malignancy, and bed-/chair-ridden status, plus an indicator for at least one of these conditions), the physician data included demographic and occupational characteristics, and practice data included factors indicating the practice type (primary care only vs. multi-specialty), practice size (total registered patients), number of physicians, panel size per physician within the practice, average annual visits per patient, and practice area socioeconomic status (SES).

### 2.3. Outcomes

The practice patterns (outcomes) data included utilization of 17 services grouped into five domains: imaging (e.g., chest X-ray, computed tomography (CT), magnetic resonance imaging (MRI), and bone scintigraphy), cardiac testing, laboratory tests (e.g., hemoglobin, thyroid-stimulating hormone (TSH), vitamin B12, prostate-specific antigen (PSA), and vitamin D), specialist consultations (pulmonology, rheumatology, and neurology), and emergency department (ED) visits (total and subcategories such as chest pain and back pain). For each service, we derived the annual count per physician–practice–year and reported rates per 1000 registered patients descriptively. We used service-specific utilization because it captures discretionary clinical decision-making at the point of care and is comparable across practices after it is standardized to panel size.

### 2.4. Surveys

To collect physician personal behavioral characteristics, during 2018–2019, we conducted face-to-face interviews with physicians in their practices. We used the following five validated scales: risk-taking scale [23], tolerance for ambiguity [24], stress due to uncertainty [25], malpractice fear scale [26], and Jefferson scale of physician empathy [19]. The risk-taking scale has six items, each rated on a 6-point Likert scale (ranging 6–36), and higher scores correspond to increased risk-taking. The tolerance for ambiguity scale includes seven items on a 6-point Likert scale (ranging 7–42), with higher scores indicating more tolerance for ambiguity. The stress due to uncertainty scale has thirteen items, each rated on a 6-point Likert scale (ranging 13–78), with higher scores corresponding to higher stress due to uncertainty. The malpractice fear scale has six items, each rated on a 5-point Likert scale (ranging 5–30,) with higher scores corresponding to increased fear of malpractice. The Jefferson scale of physician empathy consists of twenty items, with each rated on a 7-point Likert scale (ranging 20–140), which higher scores indicate higher levels of empathy.

Survey responses were collected without direct identifiers. To link surveys with utilization/covariate data from Clalit electronic health records, we used a deterministic match based on physician characteristics that were measured identically in both sources (e.g., age, sex, and core occupational attributes such as seniority and board certification), alongside the practice and year context of participation. Datasets were fully de-identified and aggregated to the physician–practice–year level.

### 2.5. Sample Size

We calculated the sample size to detect 10% of variance to be explained by the behavioral patterns, with a type I error of 0.05 and power of 0.80 to be 1000 units of the analysis [27]. In our setting, 1000 observations, each representing a year of practice of a single physician in a single practice, could be achieved by enrolling 140 physicians with an average of six years of practice times 1.2 practices per physician per annum. Therefore, we reached out to 160 physicians, estimating that more than 140 would respond.

### 2.6. Statistical Analysis

The unit was the physician–practice–year (each row = one physician at one practice in one year). For example, a physician who worked in two practices from 2011 to 2014 would have 8 rows in the dataset (four years × two practices). Since the practices differed in patient volume, for each service we computed annual rates per 1000 registered patients for descriptive reporting.

We first analyzed the patient, physician, and practice characteristics of all physicians included in the study. Quantitative variables with a normal distribution were described by the mean and standard deviation (SD), those with a non-normal distribution by the median and interquartile range (IQR), and categorical variables by the frequencies and percentages.

For each service outcome, we fitted a separate generalized linear mixed model (negative binomial, log link) for counts, with log (patient census) as an offset. Random intercepts were specified for physicians, practices, and years. We report Nakagawa’s marginal R^2^ [28,29] (fixed effects only) to quantify explained variance. To attribute explanatory power by domain, we fitted the following four parallel models per service—each with an identical outcome, link, offset, and random-effects structure—containing only one covariate block: (i) patient case mix, (ii) physician demographic/occupational, (iii) practice context, and (iv) behavioral traits (the five validated scales as a block). The marginal R^2^ from each model reflects the proportion explained by that block alone; since blocks may overlap, these values are descriptive and not additive. To summarize overall explained variance, we summed the four block-specific marginal R^2^ values; accordingly, the complement to 1 provides an approximate unexplained proportion.

We used the “glmmTMB” and “performance” R packages [29] (including Nakagawa’s R^2^ for mixed models), Version 1.0.136. Any *p*-values < 0.05 were considered significant.

### 2.7. Sensitivity Analysis

To assess potential selection bias, we first compared respondents and non-respondents across utilization and patient/physician/practice characteristics. For any characteristic showing a meaningful difference (e.g., patient census), we then restricted the analysis to study participants and formed two subgroups defined by that characteristic. Within participants, we (a) re-examined whether additional characteristics differed between the two subgroups, and (b) re-ran the primary modeling pipeline for all services to test whether the proportion of variance explained and domain contributions differed between groups.

### 2.8. Missing Data Analysis

Partially missing values in the surveys for a specific question were imputed as a mean response per physician in the relevant survey. 

### 2.9. Ethics Approval and Consent to Participate

This study was approved by the institutional ‘Helsinki’ review board committee (Soroka University Medical Center; protocol 0063-14-SOR) on 6 March 2014, valid through 5 March 2019. An extension was granted on 3 February 2019 through 4 March 2020. The board granted a waiver of informed consent for the secondary analysis of the de-identified data, and written informed consent was obtained from physicians for the survey component.

## 3. Results

### 3.1. Study Population

Out of 228 physicians meeting the inclusion criteria, we approached 160 (64%), of whom 146 (91.3%) responded. The remaining 82 non-respondents included two subgroups: 68 physicians who were not approached, primarily because they were not present at the practice on the day of the survey, and 14 physicians who were approached but declined to participate. Table 1 depicts the physician, patient, demographic, occupational, practice, and specialists’ utilization characteristics. Forty-eight percent (70) were males; the median age was 49.0 (interquartile range [IQR] 44.0–56.3) with a median length of practice of 22 years (IQR 13–31). More than half of the physicians (76) were board-certified specialists in primary care medicine. The median number of patients per physician was 1124.57 (IQR 894.21–1275.94). The annual median percentage of patients per practice with diabetes, hypertension, and malignancy was 15.69%, 26.89%, and 6.82%, respectively. Table 1 shows the patient, physician, and practice characteristics of the 146 physicians.

### 3.2. Surveys

The median score for empathy was 119 (possible range: 20–140), for tolerance, for ambiguity it was 26.0 (possible range: 7–42), for risk-taking it was 17.0 (possible range: 6–36), for fear of malpractice it was 17.0 (possible range: 5–36), and for stress due to uncertainty it was 42.0 (possible range: 13–78) (Table 2). The correlations between the scores were the highest between tolerance for ambiguity and stress due to uncertainty (Rs = 0.63, *p*-value < 0.001), followed by stress due to uncertainty and fear from malpractice (Rs = 0.49, *p*-value < 0.001), and tolerance for ambiguity and fear from malpractice (Rs = 0.43, *p*-value < 0.001) surveys. The empathy score was negatively correlated in all surveys with R’s ranging from (−0.13) for risk-taking to (−0.30) for stress due to uncertainty, with a *p*-value < 0.001 for all.

### 3.3. Scores Correlation with the Health Services Use

Physicians were ranked by order of utilization level within each health service, their average ranks calculated, and then these averages were correlated with the five questionnaires. The correlation analysis revealed that the average rank of physicians was not significantly associated with any of the measured psychological scores. Specifically, there was a negative correlation with risk-taking (r = −0.057, *p* = 0.492) and empathy (r = −0.130, *p* = 0.117). In contrast, positive correlations were observed with tolerance for ambiguity (r = 0.132, *p* = 0.112), stress due to uncertainty (r = 0.152, *p* = 0.068), and fear of malpractice (r = 0.043, *p* = 0.604).

### 3.4. Explained Variation by the Patient, Physician and Practice Domains

Table 3 presents the proportion of variation in the practice patterns explained by patient, physician (demographic and occupational vs. psychological), and practice characteristics. Patient characteristics explained 18.9% of the variation, followed by practice characteristics (10.2%), and physician demographic and occupational characteristics (6.0%).

### 3.5. Explained Variation by Physician Personality Assessment

Table 4 shows the percentage of variation explained by the surveys’ responses. The overall median explained variation was 2.3% (IQR = 1.5 − 3.3%). The median explained variation was the highest for stress from uncertainty (0.7%) and was lowest for risk aversion and empathy (0.2%). Therefore, the overall total median explained variation was 40.4% (IQR = 32.4 − 49.0%).

### 3.6. Sensitivity Analysis

Of the 228 eligible physicians, 160 (64%) were approached, and 146 (91.3%) participated; the 82 non-respondents comprised 68 physicians who were not approached (typically absent from the practice on survey day) and 14 who declined. As shown in Supplementary Table S1, respondents had ~150 more patients per practice on average and a higher utilization of several services (notably CT, MRI, cardiac testing, and bone scintigraphy), whereas most patient characteristics (age, major morbidities, etc.) were similar between respondents and non-respondents. Item-level survey absence was addressed via physician-specific mean imputation per instrument, as pre-specified.

Since practice size may influence service utilization rates, among the physicians included in the study (respondents; n = 146), we conducted a sensitivity analysis stratified by patient census. Using the analytic cohort mean (1103 patients per practice) as the threshold, physicians were classified as low-census (<1103; n = 68; mean ± SD = 796.6 ± 253.8) or high-census (≥1103; n = 78; 1370.0 ± 213.7).

As detailed in Supplementary Table S2, six services differed significantly between strata, and pulmonology consultations were higher in the low-census stratum, whereas total ED referrals, ED referrals for chest pain, chest X-rays, and the laboratory tests hemoglobin and TSH were higher in the high-census stratum. High-census practices also had more registered patients and higher area-level SES. Patient characteristics did not differ meaningfully between strata.

The variance explained by the combined behavioral scales was similar in both strata; the median (IQR)was 3.7% (2.7–6.4%) in the low-census group vs. 3.6% (2.6–4.6%) in the high-census group (*p* = 0.32; Supplementary Tables S3 and S4), indicating that results were robust to practice size differences.

## 4. Discussion

In this study, we found that a combination of patient, practice, and physician personal and behavioral characteristics explain only 40% of the variation in health services utilization in primary care. Physician behavioral characteristics assessed by the validated surveys, including risk-taking, tolerance for ambiguity, stress due to uncertainty, fear from malpractice, and empathy, explained a minor part of the variation.

Previous research has demonstrated that MPV is likely associated with factors intrinsic to individual physicians, as their practice patterns remain constant and consistent across different years and health services [6,7]. This consistency indicates that physicians who refer many, few, or a moderate number of tests do so uniformly over time and across all health services. Therefore, we believe that our hypothesis that this significant unexplained variation can be attributed to the physicians’ psychological characteristics remains valid and should not be dismissed. We propose three potential explanations for our findings and suggest corresponding directions for future investigations.

### 4.1. Tools for the Assessment of Behavioral Characteristics

The first possible explanation is related to the way in which we measured the behavioral variables. Surveys, while commonly used, may not be optimal for assessing complex behavior due to several inherent limitations and cognitive biases. Response bias is a significant issue, where participants may not accurately self-report their behaviors or attitudes, either consciously or unconsciously providing socially desirable answers [30]. Additionally, surveys with low response rates can lead to a non-response bias, where the views of those who do respond may not represent the larger population. Furthermore, surveys typically provide only a snapshot in time, lacking the ability to reflect changes in behavior over time [31]. Moreover, they often fail to capture the nuanced and dynamic nature of decision-making in clinical settings. More advanced tools than surveys can be better suited to assessing the behavioral factors, including the following: clinical vignettes, role plays [32], computer games, biomarkers, previous decisions (e.g., financial investments, MD own health decisions) [33], and peers’ ranking, etc.

### 4.2. Other Psychological Factors Can Explain MPV

Other psychological domains not assessed in the current study, such as personality traits [34] and attitudes [35], can play a significant role in MPV. Neuroticism and extroversion are linked to spontaneous decision-making, while agreeableness and conscientiousness align with intuitive and dependent styles, and openness relates to rational decision-making [36]. Furthermore, while we have focused on behavioral characteristics, a number of biases such as cognitive, affective, stereotype, and knowledge biases might also be associated with MPV.

Future research should use qualitative methods to explore these biases, by, for example, focusing on physicians with extremely low and high uses of health services to understand their decision-making processes. Identifying specific psychological characteristics or biases could validate our hypothesis and guide the development of intervention programs to address the potentially modifiable factors.

### 4.3. Can We Reduce Medical Practice Variation Without Fully Understanding Its Causes?

If previous approaches to assess physician behaviors and psychological characteristics do not sufficiently explain MPV, it may be necessary to reconsider our hypothesis and address the unexplained variation directly. While MPV is partly driven by systematic biases, a significant portion stems from noise—random inconsistencies in clinical decision-making [37]. Therefore, even without fully identifying the underlying causes, targeted interventions can still help reduce variation and improve consistency in medical practice. Implementation science provides a framework for these efforts, emphasizing the need for multilevel strategies tailored to specific barriers in each healthcare setting [38].

In practical terms, health systems can (a) focus on services showing the largest residual variation in our cohort (for example, selected imaging, ED referrals, and specific laboratory tests), (b) implement standardized diagnostic pathways and default order sets for those services [39], (c) provide regular, confidential audit-and-feedback with peer benchmarking [40], and (d) deploy lightweight, context-aware decision support to nudge toward guideline-concordant use while minimizing alert fatigue [41,42]. These actions align with our empirical results and offer a feasible route to narrowing unwarranted differences without relying on traits that, in our data, added limited explanatory power.

### 4.4. Limitations

This study has several important limitations, described in greater detail in our prior publications [6,7,8]. We did not model system-level determinants that may influence MPV—such as payment arrangements and incentives, service availability and access (e.g., distance, wait times), workflow and staffing constraints, or contemporaneous policy/guideline changes—so residual confounding by unmeasured health system factors is possible. In addition, our outcomes were service-specific utilization counts rather than measures of appropriateness or patient-oriented outcomes (e.g., hospitalizations, mortality), which limit inferences about overuse/underuse and clinical impact.

The specific limitations of this phase of our study are that the characteristics assessed by the abstract surveys may not fully represent the physicians’ action in real-life clinical practice.

In addition, despite the extended time period between the psychological characteristics assessment in 2018–2019 and the utilization data, which was obtained for years 2011–2017, we assume that the behavior characteristics and practice habits were substantially stable [43]. To align the utilization data with behaviors measured closest to the survey period, we restricted the cohort to physicians practicing throughout 2017. However, this approach also means that our data do not capture potential changes in practice patterns after 2017. While the psychological drivers of medical practice variation are unlikely to have changed significantly, external factors such as evolving clinical guidelines, healthcare policies, and system-level influences may have shaped physician decision-making in more recent years. Thus, the age of the data represents a potential limitation, but given the stability of core behavioral traits, its impact on our findings is expected to be minimal.

A somewhat not optimal response rate (36% of the eligible physicians were either not approached or refused to respond) could have resulted in a selection bias. However, our sensitivity analysis comparing characteristics of respondents with non-respondents has demonstrated that the probability of the selection bias was low.

## 5. Conclusions

In this study we have shown that key physician behavioral characteristics, including risk-taking, tolerance for ambiguity, stress due to uncertainty, fear from malpractice, and empathy explain only a minority of MPV. Moreover, sixty percent of the MPV remains unexplained even after adjusting for a variety of patient, physician and system characteristics. Therefore, before rejecting our hypothesis, we suggest either using other methodologies, besides surveys, to assess physician behavior, or a qualitative research approach, or we suggest that psychological characteristics do not explain MPV, and therefore one should focus on adjusting physicians’ practice rather than investigating its determinants.

## Figures and Tables

**Table 1:** Patient, physician, and practice characteristics for all 146 physicians.

Domain		Characteristic	N = 146
Patients		Age (median [IQR])	45.68 [36.80, 48.65]
		SES (median [IQR])	5.98 [2.69, 8.48]
		Percent married (median [IQR])	55.98 [49.13, 62.30]
		Percent males (median [IQR])	47.27 [43.43, 50.02]
		Percent Bedouin Arabs (median [IQR])	0.58 [0.14, 56.84]
		Percent with diabetes (median [IQR])	15.69 [10.94, 20.90]
		Percent with hypertension (median [IQR])	26.89 [14.24, 36.95]
		Percent bed-ridden (median [IQR])	10.94 [4.80, 14.69]
		Percent patients with malignancy (median [IQR])	6.82 [2.89, 12.90]
Physician		Age (median [IQR])	49.00 [44.00, 56.33]
		Seniority in Clalit health service (median [IQR])	12.95 [8.45, 20.40]
		Seniority (median [IQR])	22.25 [13.00, 31.00]
		Specialists in primary care, n (%)	76 (52.1)
		Born in Israel, n (%)	39 (26.7)
		Male gender, n (%)	70 (47.9)
Practices		Practice type (%)	
		Primary medicine	51 (34.9)
		Primary medicine and other specialists	78 (53.4)
		Rural	17 (11.6)
		Total physicians in the practice (median [IQR])	4.00 [2.89, 5.17]
		Total patients in practice (median [IQR])	4578.14 [3059.00, 6672.71]
		Number of patients per physician in practice (median [IQR])	1124.57 [894.21, 1275.94]
		Average number of annual visits per patient (median [IQR])	5.15 [4.46, 5.80]
		Practice SES (median [IQR])	5.74 [2.55, 7.87]
		Number of practices per physician (median [IQR])	1.00 [1.00, 1.00]
Utilizations	Other specialist	Pulmonary specialist (median [IQR])	22.87 [13.35, 33.25]
		Rheumatology specialist (median [IQR])	14.71 [9.06, 22.12]
		Neurology specialist (median [IQR])	58.03 [41.91, 76.19]
	Emergency department	Total utilizations (median [IQR])	127.85 [85.66, 189.91]
		due to Back pain (median [IQR])	3.12 [1.61, 5.09]
		Due to Chest pain (median [IQR])	3.31 [1.21, 7.66]
	Imaging/tests	Chest X-ray (median [IQR])	85.19 [57.13, 112.05]
		CT (median [IQR])	63.50 [41.10, 94.56]
		MRI (median [IQR])	16.69 [10.58, 26.21]
		Cardiac testing (median [IQR])	70.32 [45.44, 101.07]
		Bone scintigraphy (median [IQR])	10.90 [6.58, 15.28]
	Blood tests	CEA (median [IQR])	31.70 [19.82, 49.65]
		Hemoglobin (median [IQR])	1897.93 [1513.32, 2345.11]
		TSH (median [IQR])	607.42 [474.37, 735.71]
		Vitamin B12 (median [IQR])	237.78 [160.12, 360.60]
		PSA (median [IQR])	59.70 [23.17, 100.78]
		Vitamin D3 (median [IQR])	61.50 [39.84, 106.24]

Table 1 presents the patient, physician demographic, occupational, and practice characteristics for all 146 physicians. Utilization rates were calculated per year per 1000 patients: annual utilizations/patient census × 1000. SES: Socioeconomic status, CT: Computed tomography, MRI: Magnetic resonance imaging, CEA: Carcinoembryonic antigen, TSH: Thyroid-stimulating hormone, PSA: Prostate-specific antigen, and IQR: Interquartile range.

**Table 2:** Survey responses for 146 physicians.

Personal Behavioral Characteristics	Risk-taking (median [IQR])	17.0 [7.00, 34.0]
	Tolerance for ambiguity (median [IQR])	26.0 [7.00, 42.0]
	Stress due to uncertainty (median [IQR])	42.0 [13.0, 77.0]
	Fear from malpractice (median [IQR])	17.0 [6.00, 30.0]
	Jefferson Scale of Physician Empathy (median [IQR])	119 [75.0, 140]

Table 2 presents the descriptives of the questionnaires’ scores. (31, 25, 26, 27, 20) IQR: interquartile range.

**Table 3:** Percent of variation explained by patient, physician, practice and personal behavioral domains.

		Patient	Physician Demographic and Occupational	Practice	Physician Personal Behavior (Surveys)	All
Specialist Visits	Pulmonary	19.9%	4.9%	6.3%	3.2%	34.3%
	Rheumatology	13.7%	11.6%	9.5%	5.6%	40.4%
	Neurology	8.3%	15.2%	9.7%	5.2%	38.4%
Emergency Department Utilizations	Total utilizations	17.2%	8.5%	20.1%	10.4%	56.2%
	Chest pain	7.2%	5.3%	4.8%	2.3%	19.6%
	Back pain	18.9%	2.5%	20.6%	4.6%	46.6%
Imaging	Chest X-ray	27.7%	9.0%	11.9%	1.7%	50.3%
	CT	15.8%	4.5%	10.4%	1.7%	32.4%
	MRI	17.0%	6.0%	4.5%	1.4%	28.9%
	Cardiac testing	10.0%	6.0%	4.5%	3.3%	23.8%
	Bone scintigraphy	10.0%	6.6%	3.3%	3.1%	23.0%
Laboratory Tests	CEA	42.4%	3.8%	9.5%	1.1%	56.8%
	Hemoglobin	24.6%	5.1%	3.3%	1.5%	34.5%
	TSH	24.9%	4.2%	11.3%	1.0%	41.4%
	Vitamin B12	24.5%	8.1%	14.6%	1.4%	48.6%
	PSA	28.9%	5.4%	12.9%	1.9%	49.1%
	Vitamin D3	22.0%	7.2%	16.8%	3.0%	49.0%
	Median (IQR)	18.9% (13.7–24.6%)	6.0% (4.9–8.1%)	9.7% (4.8–12.9%)	2.3% (1.5–3.3%)	40.4% (32.4–49.0%)

Table 3 is a heat map showing the variation explained by the patient, physician (demographic and occupational vs. psychological), and practice characteristics. The darker the red color of the cell, the greater the explained variation. To calculate the explained variation, we used Nakagawa’s R^2^ for mixed models, computing the marginal r-squared, reflecting the percentage of variation explained by the domains. CT: Computed tomography, MRI: Magnetic resonance imaging, CEA: Carcinoembryonic antigen, TSH: Thyroid-stimulating hormone, PSA: Prostate-specific antigen, and IQR: Interquartile range.

**Table 4:** Percent of variation explained by the physicians’ personal behavioral characteristics.

		Risk-Taking Scale	Tolerance for Ambiguity	Stress Due to Uncertainty	Fear from Malpractice	Jefferson Scale of Physician	All
Specialist	Pulmonary	0.00%	0.50%	1.10%	0.00%	2.10%	3.20%
Visits	Rheumatology	0.80%	1.70%	2.40%	0.30%	0.30%	5.60%
	Neurology	0.00%	1.40%	1.50%	1.10%	4.20%	5.20%
Emergency department Visits	Total visits	0.70%	3.10%	3.60%	2.10%	7.00%	10.40%
	Chest pain	0.00%	0.50%	1.50%	1.60%	0.30%	2.30%
	Back pain	0.20%	0.70%	0.80%	1.40%	2.20%	4.60%
Imaging	Chest X-ray	0.20%	0.80%	0.50%	0.10%	0.20%	1.70%
	CT	0.20%	0.40%	0.70%	0.10%	0.20%	1.70%
	MRI	0.50%	0.20%	0.50%	0.00%	0.20%	1.40%
	Cardiac testing	0.30%	0.60%	0.60%	0.60%	0.00%	3.30%
	Bone scintigraphy	0.10%	1.70%	1.80%	0.00%	0.10%	3.10%
Laboratory	CEA	0.00%	0.10%	0.10%	1.00%	0.00%	1.10%
Tests	Hemoglobin	0.40%	0.50%	0.00%	0.00%	0.10%	1.50%
	TSH	0.00%	0.00%	0.40%	0.40%	0.00%	1.00%
	Vitamin B12	0.70%	0.00%	0.00%	0.00%	0.50%	1.40%
	PSA	0.00%	0.00%	0.10%	0.80%	0.00%	1.90%
	Vitamin D3	2.20%	0.00%	0.00%	0.60%	0.20%	3.00%
	Median (IQR)	0.20% (0.0–0.5%)	0.50% (0.2–0.8%)	0.65% (0.3–1.5%)	0.35% (0.0–1.0%)	0.20% (0.1–0.5%)	2.30% (1.5–3.3%)

Table 4 is a heat map showing the variation explained by the surveys separately and together. The darker the red color of the cell, the greater the explained variation. To calculate the explained variation, we used Nakagawa’s R^2^ for mixed models, computing the marginal r-squared, reflecting the percentage of variation explained by the domains. CT: Computed tomography, MRI: Magnetic resonance imaging, CEA: Carcinoembryonic antigen, TSH: Thyroid-stimulating hormone, PSA: Prostate-specific antigen, and IQR: Interquartile range.

## Data Availability

The datasets used and/or analyzed during the current study are available following local Ethics Committee approval. Please contact Sagi Shashar shasharsagi1@gmail.com.

## Supplementary Materials

The following supporting information can be downloaded at: https://www.mdpi.com/article/10.3390/jcm14207396/s1. Table S1: Sensitivity Analysis—Comparison between Physicians Who Answered the Questionnaire and Those Who Did not; Table S2: Sensitivity Analysis—Comparison between Physicians with high vs. low patient census; Table S3: Sensitivity Analysis—Explained Variance between Physicians with Lower Patient Census; Table S4: Sensitivity Analysis –Explained Variance between Physicians with Higher Patient Census.

## Abbreviations

MPV	Medical practice variation
SES	Socioeconomic status
CT	Computed tomography
MRI	Magnetic resonance imaging
TSH	Thyroid-stimulating hormone
CEA	Carcinoembryonic antigen
PSA	Prostate-specific antigen
SD	Standard deviation
IQR	Interquartile range
