# Supplementary material for: The Hidden Influences: Psychological Drivers of Medical Practice Variation
Source: J Clin Med. 2025 Oct 20;14(20):7396. doi: 10.3390/jcm14207396 (PMC12565173; doi:10.3390/jcm14207396)
Supplement: Supplementary file 1 [file jcm-14-07396-s001.zip › jcm-3855180-supplementary.pdf]

Table 1s: Sensitivity Analysis – Comparison between Physicians Who Answered the Questionnaire and Those Who Did not

		Did not answer	Answered	P-value
	n	82	146	
Referrals	Pulmonary specialist (median [IQR])	22.53 [13.59, 40.48]	22.87 [13.35, 33.25]	0.565
	Rheumatology specialist (median [IQR])	12.24 [6.84, 21.65]	14.71 [9.06, 22.12]	0.186
	Neurology specialist (median [IQR])	52.52 [30.94, 80.00]	58.03 [41.91, 76.19]	0.283
	CT (median [IQR])	54.48 [28.32, 81.92]	63.50 [41.10, 94.56]	**0.039**
	Total referrals to ED (median [IQR])	115.82 [75.33, 178.83]	127.85 [85.66, 189.91]	0.221
	Referrals to ED due to Back pain (median [IQR])	2.29 [1.30, 4.18]	3.12 [1.61, 5.09]	0.075
	Referrals to ED due to Chest pain (median [IQR])	2.21 [0.52, 5.82]	3.31 [1.21, 7.66]	0.066
	Chest X ray (median [IQR])	86.04 [42.75, 126.36]	85.19 [57.13, 112.05]	0.518
	MRI (median [IQR])	13.66 [5.78, 19.00]	16.69 [10.58, 26.21]	**<0.001**
	Cardiac testing (median [IQR])	57.51 [22.69, 97.63]	70.32 [45.44, 101.07]	**0.037**
	Bone scintigraphy (median [IQR])	7.48 [3.65, 13.39]	10.90 [6.58, 15.28]	**0.005**
	CEA (median [IQR])	27.67 [12.41, 51.83]	31.70 [19.82, 49.65]	0.211
	HGB (median [IQR])	1534.05 [1153.89, 1990.90]	1897.93 [1513.32, 2345.11]	**0.003**
	TSH (median [IQR])	40.98 [9.45, 84.14]	52.37 [18.82, 87.79]	0.231
	B12 (median [IQR])	640.31 [485.99, 802.70]	607.42 [474.37, 735.71]	0.671
	PSA (median [IQR])	250.92 [145.27, 385.63]	237.78 [160.12, 360.60]	0.95
	D3 (median [IQR])	55.49 [13.97, 110.68]	59.70 [23.17, 100.78]	0.857
Practices	Practice type (%)			
	Primary medicine	68.58 [35.73, 120.65]	61.50 [39.84, 106.24]	0.935
	Primary medicine and specialists	38 (46.3)	51 (34.9)	
	Rural	35 (42.7)	78 (53.4)	
	Total physicians in practice (median [IQR])	9 (11.0)	17 (11.6)	0.224
	Total patients in practice (median [IQR])	4207.74 [1138.60, 6555.00]	4578.14 [3059.00, 6672.71]	0.096
	Number of patients per physician in practice (median [IQR])	1011.44 [715.25, 1267.38]	1124.57 [894.21, 1275.94]	0.07
	Average number of annual visits per patient (median [IQR])	5.08 [4.27, 5.82]	5.15 [4.46, 5.80]	0.668
	SES (median [IQR])	5.56 [1.46, 7.56]	5.74 [2.55, 7.87]	0.159
	Number of practices per physician (median [IQR])	1.00 [1.00, 2.00]	1.00 [1.00, 1.00]	0.666

Patients	Patient census (median [IQR])	988.54 [660.34, 1300.18]	1135.21 [907.89, 1358.57]	**0.041**
	Age (median [IQR])	45.56 [34.43, 49.38]	45.68 [36.80, 48.65]	0.962
	SES (median [IQR])	6.16 [1.47, 8.31]	5.98 [2.69, 8.48]	0.211
	Percent Bedouin Arabs (median [IQR])	0.56 [0.12, 81.56]	0.58 [0.14, 56.84]	0.937
	Percent with diabetes (median [IQR])	16.36 [9.16, 20.60]	15.69 [10.94, 20.90]	0.433
	Percent with blood pressure (median [IQR])	27.33 [10.56, 37.15]	26.89 [14.24, 36.95]	0.465
	Percent bed/chair ridden (median [IQR])	10.37 [3.10, 14.73]	10.94 [4.80, 14.69]	0.501
	Percent patients with malignancy (median [IQR])	5.94 [2.23, 13.76]	6.82 [2.89, 12.90]	0.597
	Percent married (median [IQR])	56.47 [48.71, 61.56]	55.98 [49.13, 62.30]	0.82
	Percent males (median [IQR])	46.27 [43.05, 50.55]	47.27 [43.43, 50.02]	0.774
Physician	Age (median [IQR])	52.25 [43.98, 57.00]	49.00 [44.00, 56.33]	0.196
	Seniority in Clalit health service (median [IQR])	14.05 [9.55, 19.85]	12.95 [8.45, 20.40]	0.546
	Seniority (median [IQR])	26.00 [14.00, 31.00]	22.25 [13.00, 31.00]	0.502
	Specialists in primary care, n (%)	36 (43.9)	76 (52.1)	0.361
	Born in Israel, n (%)	25 (30.5)	39 (26.7)	0.649
	Male gender, n (%)	44 (53.7)	70 (47.9)	0.49

Table 2s: Sensitivity Analysis – Comparison between Physicians with high vs. low patient census

		Low patient census	High patient census	P-value
	n	68	78	
Referrals	Pulmonary specialist (median [IQR])	27.24 [17.83, 37.43]	16.41 [9.49, 26.67]	**<0.001**
	Rheumatology specialist (median [IQR])	14.77 [8.03, 21.87]	14.44 [9.85, 23.61]	0.2
	Neurology specialist (median [IQR])	54.41 [38.99, 70.96]	60.14 [46.69, 80.05]	0.184
	CT (median [IQR])	62.09 [39.01, 87.70]	67.68 [43.25, 103.84]	0.26
	Total referrals to ED (median [IQR])	98.42 [60.98, 172.74]	150.63 [118.55, 193.08]	**0.001**
	Referrals to ED due to Back pain (median [IQR])	2.26 [1.29, 4.38]	3.82 [2.43, 5.43]	**0.001**
	Referrals to ED due to Chest pain (median [IQR])	1.86 [0.65, 3.64]	5.57 [2.58, 10.21]	**<0.001**
	Chest X ray (median [IQR])	77.61 [52.06, 100.56]	92.20 [61.75, 126.88]	**0.038**
	MRI (median [IQR])	17.03 [10.43, 27.30]	16.39 [10.76, 23.09]	0.646
	Cardiac testing (median [IQR])	69.04 [46.62, 100.11]	71.04 [45.44, 101.07]	0.981
	Bone scintigraphy (median [IQR])	11.95 [7.03, 15.73]	10.24 [6.43, 13.97]	0.426
	CEA (median [IQR])	35.82 [21.41, 49.48]	28.05 [18.01, 49.58]	0.444
	HGB (median [IQR])	1684.50 [997.68, 1911.75]	2227.39 [1814.50, 2542.66]	**<0.001**
	TSH (median [IQR])	34.23 [14.93, 62.82]	71.25 [43.34, 120.70]	**<0.001**
	B12 (median [IQR])	604.93 [494.00, 729.71]	610.33 [465.39, 759.58]	0.772
	PSA (median [IQR])	240.23 [169.09, 356.83]	237.78 [160.12, 364.02]	0.978
	D3 (median [IQR])	54.61 [20.03, 96.70]	59.97 [28.40, 103.92]	0.437
Practices	Practice type (%)			
	Primary medicine	21 (30.9)	30 (38.5)	
	Primary medicine and specialists	32 (47.1)	46 (59.0)	
	Rural	15 (22.1)	2 (2.6)	
	Total patients in practice (median [IQR])	3136.31 [1955.46, 5046.04]	5659.26 [4274.57, 6916.64]	**<0.001**
	Average number of annual visits per patient (median [IQR])	5.15 [4.57, 5.83]	5.13 [4.43, 5.69]	0.778
	SES (median [IQR])	3.87 [1.53, 7.57]	6.64 [2.94, 8.24]	**0.008**
	Number of practices per physician (median [IQR])	1.00 [1.00, 2.00]	1.00 [1.00, 1.00]	**0.004**
	Patient census (median [IQR])	904.86 [671.73, 979.46]	1350.92 [1214.79, 1446.09]	**<0.001**
Patients	Age (median [IQR])	45.68 [36.46, 48.46]	45.91 [37.68, 48.88]	0.817
	SES (median [IQR])	3.92 [1.57, 7.86]	6.89 [3.09, 8.84]	0.011

	Percent Bedouin Arabs (median [IQR])	0.78 [0.14, 63.29]	0.47 [0.14, 47.35]	0.754
	Percent with diabetes (median [IQR])	15.01 [10.45, 18.79]	18.29 [11.92, 22.11]	0.137
	Percent with blood pressure (median [IQR])	25.46 [13.81, 35.01]	28.33 [14.64, 37.33]	0.401
	Percent bed ridden (median [IQR])	10.82 [4.99, 14.59]	10.96 [4.64, 14.79]	0.913
	Percent patients with malignancy (median [IQR])	5.42 [2.29, 11.41]	8.04 [4.32, 13.39]	0.065
	Percent with at least one of the above diagnoses (median [IQR])	32.58 [14.56, 46.31]	30.61 [17.52, 44.76]	0.981
	Percent married (median [IQR])	58.79 [51.00, 63.67]	54.67 [47.81, 61.05]	0.068
	Percent males (median [IQR])	47.26 [43.20, 49.81]	47.34 [43.98, 50.64]	0.533
Physician	Age (median [IQR])	53.00 [46.54, 57.25]	48.00 [42.00, 53.38]	0.006
	Seniority in Clalit health service (median [IQR])	16.35 [10.00, 23.22]	11.65 [7.30, 18.55]	0.004
	Seniority (median [IQR])	26.38 [16.00, 32.00]	19.75 [12.00, 28.38]	0.017
	Specialists in primary care, n (%)	41 (60.3)	37 (47.5)	0.298
	Born in Israel, n (%)	19 (27.9)	20 (25.6)	0.9
	Male gender, n (%)	32 (47.1)	38 (48.7)	0.973

Table 3s: Sensitivity Analysis –Explained Variance between Physicians with Lower Patient Census

		RTS	TA	SUS	FMS	JSE	ALL
Specialist Visits	Pulmonary	1.00%	2.00%	5.30%	3.60%	6.80%	10.10%
	Rheumatology	0.00%	2.50%	2.90%	1.30%	4.80%	6.40%
	Neurology	0. 2%	3.00%	5.90%	2.80%	8.80%	11.60%
ED Referrals	Total referrals	0.50%	1.10%	11.10%	5.30%	10.70%	18.10%
	Chest pain	0.00%	0.40%	1.20%	3.20%	0.30%	3.50%
	Back pain	0.40%	3.10%	3.40%	3.50%	3.20%	6.90%
Imaging	Chest X ray	0.60%	0.70%	0.40%	0.00%	1.60%	2.70%
	CT	1.30%	0.00%	0.70%	0.10%	0.10%	2.70%
	MRI	2.80%	0.70%	0.40%	0.10%	0.10%	3.60%
	Cardiac testing	1.40%	0.10%	0.10%	0.10%	0.00%	1.90%
	Bone scintigraphy	0.00%	2.00%	1.80%	0.00%	0.10%	3.20%
Laboratory Tests	CEA	0.00%	2.00%	0.90%	1.00%	0.00%	2.50%
	HGB	0.20%	2.40%	3.70%	5.60%	2.20%	7.10%
	TSH	0.20%	0.60%	0.30%	0.20%	0.80%	2.50%
	Vitamin B12	0.80%	3.50%	2.50%	0.90%	0.40%	4.60%
	PSA	0.00%	1.20%	0.10%	0.30%	0.60%	3.70%
	Vitamin D3	2.80%	0.40%	0.70%	2.20%	1.40%	4.80%
	Median(IQR)	0.5% (0.0%-1.3%)	1.2% (0.6%-2.4%)	1.2% (0.4%-2.9%)	1.0% (0.1%-2.8%)	0.8% (0.1%-2.5%)	3.7% (2.7%-6.4%)

Risk-Taking Scale (RTS) Tolerance for Ambiguity (TA), Stress due to Uncertainty Scale (SUS), Fear of Malpractice Scale (FMS), Jefferson Scale of Physician Empathy (JSE)

Table 4s: Sensitivity Analysis –Explained Variance between Physicians with Higher Patient Census

		RTS	TA	SUS	FMS	JSE	ALL
Specialist Visits	Pulmonary	1.10%	0.00%	0.10%	1.40%	0.00%	3.30%
	Rheumatology	3.10%	1.40%	1.40%	0.10%	0.30%	5.10%
	Neurology	0. 4%	1.10%	0.10%	0.60%	0.40%	2.60%
ED Referrals	Total referrals	0.30%	0.30%	0.60%	1.40%	0.70%	2.40%
	Chest pain	0.00%	0.70%	1.90%	0.20%	0.10%	2.00%
	Back pain	0.50%	0.80%	1.00%	3.60%	2.60%	6.70%
Imaging	Chest X ray	0.10%	1.20%	0.10%	0.00%	2.00%	4.70%
	CT	0.00%	2.70%	1.40%	0.00%	0.00%	3.80%
	MRI	0.00%	1.80%	1.70%	0.10%	0.10%	2.80%
	Cardiac testing	0.10%	1.30%	1.20%	0.80%	0.30%	5.90%
	Bone scintigraphy	0.20%	4.70%	1.90%	0.00%	0.20%	7.40%
Laboratory Tests	CEA	0.00%	1.00%	0.00%	0.40%	0.20%	3.80%
	HGB	0.40%	1.10%	0.00%	0.40%	0.10%	1.90%
	TSH	0.00%	1.10%	0.10%	0.10%	0.00%	2.20%
	Vitamin B12	1.10%	2.10%	0.90%	0.20%	0.00%	3.70%
	PSA	0.00%	0.70%	0.40%	0.70%	0.00%	3.60%
	Vitamin D3	1.40%	0.60%	1.10%	0.00%	1.30%	3.30%
	Median(IQR)	0.2% (0.0%-0.7%)	1.1% (0.7%-1.4%)	0.9% (0.1%-1.4%)	0.2% (0.1%-0.6%)	0.2% (0.0%-0.5%)	3.6% (2.6%-4.6%)

Risk-Taking Scale (RTS) Tolerance for Ambiguity (TA), Stress due to Uncertainty Scale (SUS), Fear of Malpractice Scale (FMS), Jefferson Scale of Physician Empathy (JSE)
